# A cross-sectional survey on the relationship between workplace psychological violence and empathy among Chinese nurses: the mediation role of resilience

**DOI:** 10.1186/s12912-024-01734-1

**Published:** 2024-02-01

**Authors:** Li Li, Xiaoli Liao, Juan Ni

**Affiliations:** 1 Department of Urology, the First Affiliated Hospital of Xinjiang Medical University, Urumqi, Xinjiang Province China; 2 Clinical Nursing Teaching and Research Section, The Second Xiangya Hospital, Central South University, Changsha, Hunan Province China; 3 Hunan Traditional Chinese Medical College, ZhuZhou, Hunan Province China

**Keywords:** Empathy, Nurse, Psychological violence, Resilience, Workplace violence

## Abstract

**Background:**

Workplace violence is one of the most serious public health issues worldwide in healthcare occupations, nurse is a profession which faces the greatest risk of exposure to workplace violence among healthcare occupations.

**Objective:**

The present study aimed to explore the relationship between workplace psychological violence and empathy among Chinese nurses, and further examine the mediation role of resilience in this relationship.

**Method:**

A cross-sectional survey was conducted among a convenience sample of clinical registered nurses in Xinjiang China from 29 September 2023 to 19 October 2023.The online questionnaire, contained the general information form, the Workplace Psychologically Violent Behaviors Instrument, the Jefferson Scale of Empathy-Healthcare Professionals Version, and the Connor-Davidson Resilience Scale, was used to collect data. The IBM SPSS statistics software version 22.0 was used to perform data analyses in forms of descriptive statistics, correlation analysis, and mediation analysis.

**Result:**

This survey recruited a convenience sample of 1613 clinical registered nurses aged 22 to 55 years who come from diverse ethnicities and worked in different departments. A total of 534 nurse experienced psychological violent, which yielded a positive rate of 33.1% for psychological violent among nurses. Pearson analysis reported a negative correlation between psychological violences and empathy (*r*=-0.724, *P* < 0.01) as well as a negative correlation between psychological violences and resilience (*r*=-0.681, *P* < 0.01). Mediation analysis reported that resilience mediated the negative relationship between psychological violence and empathy, the mediation effect accounted for ab/(ab + c’) = 23.40% of the total effect.

**Conclusion:**

This study supported an inverse ralationship between psychological violence and empathy among Chinese nurses where resilience acted as a protective factor to mediated the negative impacts of psychological violences on empathy These results directed health policies and clinical interventions to equip nurses with resilience to copy with and recover from workplace psychological violence.

## Background

Workplace violence refers to incidents where staff members are abused, intimidated, or assaulted under work circumstances [1], which occurs in the forms of physical violence and psychological violence [2]. Workplace psychological violence refers to a kind of psychological terrors in the forms of verbal abuses, insults, threats, attacks, and tortures, which damages physical, mental, spiritual, moral, and social functions of the victims [3].The International Labor Organization (ILO) reported that healthcare occupation represented a profession confronted the highest risk of exposure to workplace violences [4, 5]. Workplace psychological violence constitutes an occupational hazard and public health issue cross borders, occupations and environments, which attracts attentions from organizations, researchers and medias around the word [6, 7]. Studies converge to support a high prevalence of workplace psychological violences across occupations and industries with fluctuations emerge across different medical systems and national conditions. An umbrella review of meta-analyses examined the prevalence of workplace violences against healthcare workers, which included 14 meta-analyses and involved 674,266 healthcare workers, found that the overall prevalence of workplace violences was 58.7%, the prevalence of physical violences was 20.8%, the prevalence of verbal violences was 66.8%, and the prevalence of sexual harassments was 10.5% [8]. A cross-sectional study investigated the prevalence of workplace violences against healthcare workers during the COVID-19 Pandemic in Israel, which adopted online questionnaires to examined different forms of workplace violences among a total of 486 healthcare workers over the past 6 months, found that 71% of respondents were exposed to workplace violences, 69% of respondents were exposed to verbal or psychological violences, and 11% of respondents were exposed to physical violences [9]. Studies emerge to emphasize the widespread prevalence of workplace psychological violences among healthcare works from Chinese healthcare systems. A cross-sectional study examined the prevalence of workplace violences towards healthcare workers in China, an international survey questionnaire was distributed among 1028 healthcare workers in a Chinese secondary hospital, found that 5.45% of respondents encountered physical violences while 41.63% experienced psychological violences in the past 12 months [10]. Workplace psychological violence exposure not only impairs the physical status and psychological conditions of healthcare workers, but also job performance and work engagement of healthcare workers [11, 12].

Workplace psychological violence represents one of the grave public health issues across occupations and industries worldwide with detrimental consequences for individuals and organizations. Nurse represents the healthcare profession which faces the highest risk of exposure to workplace psychological violences among healthcare workers [13]. Nurses encounter workplace psychological violences from different perpetrators in terms of colleagues, managers, and patients. A quantitative review examined the prevalence of workplace violences among nurses which included 136 original articles and involved 151,347 nurses, reported a prevalence of 36.4% for physical violences and a prevalence of 66.9% for psychological violences among nurses over the past year [14]. A recent meta-analysis explored the prevalence of workplace violences among Chinese nurses which included 38 original studies and involved 22,968 nurses, found that 67-75% of nurses experienced workplace violences, 11-18% of nurses experienced physical violences, 58-67% of nurses experienced verbal abuses, and 39-48% of nurses experienced threats [15]. Workplace psychological violence constitutes a topic of public health issue and academic research attention, which not only impacts professional performance and workplace resource but also impairs psychological capital and emotional reserve. Previous studies converged to confirm the adverse consequences of workplace psychological violences against nurses, which included decreased job satisfaction and increased psychological symptom as well as decreased medical service quality and increased human resource cost [16]. The high prevalence and serious implication of workplace psychological violence for nurses and hospitals require further characterization of the destructive impacts and identification of the protective factors.

Empathy refers to a cognitive attribute and psychological competency which enables individuals to sense and perceive the thoughts, intentions, and motivations of others [17]. Nurses who experience workplace psychological violences could reduce work passion and impair empathy ability, which provides a clue to the mechanism of how workplace psychological violences impact nurse outcomes. Previous studies found that the experience of workplace incivility decreased empathy ability and elicited empathy fatigue of victimized nurses [18]. Resilience refers to a personality trait and psychological resource which enables individual to cope with and rebound from failure or adversity [19]. Resilience constitutes a complex and dynamic process among which nurses adopt problem solving skills to address workplace adversity and develop coping strategies to minimize psychological distress [20]. Previous studies found that resilience constituted a personal resource which alleviated the adverse impacts of workplace psychological violence and maintained the mental health of affected nurses [21]. The National Health Commission of China in 2015 established the “Safe Hospital” policy to build a safe work environment for healthcare workers [22], which urges the identification of protective factors and risk factors for safe work environment in healthcare systems.

Most studies investigated the incidence and the impact of workplace violences among healthcare workers in general, few studies examined the prevalence and consequence of psychological violences among nurses in particular. Furthermore, previous studies mainly focused on the effects of workplace violences on psychosocial health indicators and work indicators, relatively little attention devoted to the impacts of psychological violences on empathy and resilience as well as the correlations of psychological violences and empathy and resilience.

**1.1 Aim**.

The present study aimed to explore the correlations of workplace psychological violences, empathy, and resilience among nurses, test the mediation effect of resilience on the relationship between psychological violence and empathy among nurses, thus provide theoretical references for hospital management and intervention formulation to resist the destructive impacts of workplace psychological violences on nurses.

## Method

The present study was reported in accordance with the Strengthening the Reporting of Observational Studies in Epidemiology (STROBE) checklist for cross-sectional studies [23] (Appendix 1).

### Design

The present study was a cross-sectional survey which was conducted among a convenience sample of clinical registered nurses in Xinjiang province China during the period of September 2023 and October 2023.

### Setting and sample

The present study adopted a convenience sampling method to recruit clinical registered nurses in The First Affiliated Hospital of Xinjiang Medical University, Urumqi, Xinjiang Province, China. The National Bureau of Statistics in 2022 reported that the total number of registered nurses in China was 5.2242 million, the number of registered nurses in Xinjiang was 89,000 composed 1.7% of registered nurses nationwide. The hospital was founded in 1954 which is the first grade-III class A hospital in Xinjiang. The hospital is the medical center of Xinjiang which has 2371 registered nurses and 2700 open beds.

Inclusion criteria were set as: nurses who acquired a Chinese registered nurse license within the valid registration period; nurses who engaged in clinical patient care with work experience of at least one year; nurses who were regular employees of the hospital; nurses who provided informed consent for voluntary participation.

Exclusion criteria were set as: non-hospital nurses, such as training nurses and nursing interns; absent nurses, such as sick leave, personal leave, and further study; non-clinical nurses, such as nurses worked in service departments or supply departments.

### Sample size

The formula N = Z_α/2_^2^π(1-π)/δ^2^ was used to calculate the sample size of this cross-sectional survey. The type I error α was set as 0.05, the Z_α/2_ was set as 1.96, and the absolute error δ was set as 0.03.π = 50% was set for calculation in order to ensure sufficient size for cross-sectional study [24]. A sample size of 1067 was derived, considering a 20% of invalid questionnaires, a minimum sample size of 1280 was required. Therefore, the recruitment of 1280 participants was sufficient to meet the sample size requirements of this cross-sectional survey.

### Recruitment

Wenjuanxing (https://www.wjx.cn/), a professional online questionnaire platform in China, was utilized to create the electronic questionnaire [25]. The social media app WeChat, a popular social communication application in China, was used to distribute the online questionnaire. All questionnaire items were imported into Wenjuanxing, which generated a website link and quick response code to access questionnaire. The research team contacted the directors of occupational department and obtained the investigation permission from the management department. The trained investigators were responsible for clarifying the purpose, content, and requirements of the investigation to participants. The trained investigators sent the website link and quick response code of the online questionnaire to nurses via WeChat, and nurses clicked the link or scanned the quick response code via mobile phones to access questionnaires. The investigated nurses were guaranteed anonymity of their responses, confidentiality of their information, and the voluntary nature of their participations. Questionnaire which submitted in less than 3 min or more than 40 min were considered invalid.

### Variables and measurements

The online questionnaire contained a general information form, the Workplace Psychologically Violent Behaviors Instrument, the Jefferson Scale of Empathy-Healthcare Professionals Version, and the Connor-Davidson Resilience Scale.

#### The general information form

A general information form was developed to obtain demographic information, which included gender, ethnicity, age, work years, educational level, occupational department, and professional title.

Gender was categorized as “Male” and “Female”. Ethnicity was categorized as “Han nationality”, “Weiwuer nationality”, “Hui nationality”, and “Other nationality”. Educational level was divided into three levels in terms of “College and lower”, “Undergraduate”, and “Master and above”. Occupational department was categorized as “Internal medicine department”, “Surgery department”, “Emergency medicine department”, and “Intensive care unit department”. Professional title was classified into three levels in terms of “Primary title”, “Intermediate title”, and “Senior title”.

#### Workplace Psychologically Violent Behaviors Instrument

The Workplace Psychologically Violent Behaviors Instrument (WPVBI) was used to measure workplace psychological violences among nurses [26, 27]. The WPVBI scale was developed by Dilek and Aytolan [17], and translated and revised by Xu et al. [27]. The WPVBI scale consisted of 32 items and 4 dimensions in terms of isolation from work (11 items), attacks on professional status (9 items), attacks on personality (9 items), and direct negative behavior (3 items). Each item was rated on a 6-point Likert-type scale ranged from 0 “never happened” to 5 “always happened”. The participants were asked to rate each item with reference to their experience in the past year prior to the data collection. The total score (ranged from 0 to 160) of the scale was the sum of the responses of each item, where higher scores indicated more workplace psychologically violence. A mean score of ≥ 1 for all 32 items on the scale (mean score = total score/32) indicated positive for psychological violence. The Cronbach’s α coefficient for the scale was 0.96, and the α coefficients for the dimensions were 0.85–0.94 [28]. The Cronbach’s α coefficient of the scale in this study was 0.96.

#### Jefferson scale of Empathy-Healthcare Professionals Version

The Jefferson Scale of Empathy-Healthcare Professionals Version (JSE-HP) was used to measure empathy among nurses [29]. The JSE-HP scale consisted of 20 items and 3 dimensions in terms of perspective-taking (10 items), compassionate care (7 items), and standing in patient’s shoes (3 items). The scale contained 10 positively scored items and 10 negatively scored items. Each item was answered on a 7-point Likert-type scale, the positively scored items were rated from 1“strongly disagree” to 7 “strongly agree” and the negatively scored items were rated from 1“strongly agree” to 7 “strongly disagree”. The sum score (ranged from 20 to 140) of the scale was the sum of the responses of each item, where higher score indicated greater empathy. The modified scale was validated and translated into Chinese, the Cronbach’s α coefficient for the scale was 0.80 and the split-half reliability coefficients of this scale was 0.79 [30]. The Cronbach’s α coefficient of the scale in this study was 0.86.

#### Connor-Davidson Resilience Scale

The Connor-Davidson Resilience Scale (CD-RISC) was a self-administered questionnaire to measure psychological resilience among nurses [31]. The CD-RISC was developed by Connor and Davidson [31], and translated and revised by Yu and Zhang [32]. The CD-RISC scale consisted of 25 self-rated items and 3 dimensions in terms of tenacity, strength, and optimism. Each items rated a 5-point Likert-type scale from 0 “not true at all” to 4 “true nearly all the time”. The participants were asked to rate each item with reference to their experience in the past month prior to the data collection. The total score of the scale was the sum of the responses of each item, where higher scores indicated higher resilience capacity. The Cronbach’s α coefficient of the total scale was 0.89, and the reliability coefficient of the Chinese version was 0.91 [33]. The Cronbach’s α coefficient of the scale in this study was 0.93.

### Data analysis

The IBM SPSS statistics software version 22.0 (IBM Corp., Armonk, NY, USA) was used to perform data analysis in terms of descriptive statistics, correlation analysis, and mediation analysis. All of the tests were two-tailed and a *p*-value of 0.05 was considered statistically significant. Descriptive analysis was performed to calculate means and standard deviation for quantitative data and frequency and percentage for qualitative data. Pearson correlation analysis was performed the explore the relationship between psychological violence, empathy, and resilience. The SPSS PROCESS Macro proposed by Hayes (2012) was used to perform mediation analysis, which examined the mediation role of resilience between psychological violence and empathy [34]. Bootstrap method was adopted to test the significance of the mediation role, bootstrap 95% confidence interval did not contain 0 indicated that mediation analysis was statistically significant. The proportion (%) of mediation effects was equal to the ratio of the mediation effect value to the total effect value which was the sum of the direct effect value and the mediation effect value.

### Ethical considerations

This study received ethic approval from the institutional review board of the hospital before data collection began (NO: K202309-41). All procedures were conducted in accordance with the provisions of the Declaration of Helsinki. The research team obtained permission from the hospital managers and human resources departments after provided information regarding the purpose and procedures of the investigation. The written informed consent was not obtained from participant because of the anonymous survey approach. The oral informed consent was approved by the institutional review board and provided by the participants. An informed consent form was presented on the cover of the online questionnaire. Participants were deemed to have given consent to participate in the survey when they returned their questionnaires. All participants consciously and voluntarily provided their consent to participate in the survey. Participants were full informed that they could withdraw at any stage of the investigation without any negative consequence. All the data collected from the participants were kept anonymous and confidential to protect their privacy.

## Result

### Demographic characteristics of participants

Table 1 presented the general characteristics of clinical registered nurses recruited in this survey. The online survey retracted 1659 questionnaires, which included 5 questionnaires with contradictory answer on age and work year, 9 questionnaires with less than 3 min response time, 15 questionnaires with identical answers, and 17 questionnaires with zigzag shape answers. The removal of 46 invalid questionnaires yielded a final of 1613 valid questionnaires for effective rate of 97.23%. This survey recruited a total of 1613 clinical registered nurses with a mean age of (32.78 ± 14.03) (as showed in Table 1).

**Table 1:** The general characteristics of participants (*n* = 1613)

Item	Category	n	%
Gender	Male	90	5.58
	Female	1523	94.42
Ethnicity	Han nationality	884	54.80
	Weiwuer nationality	502	31.12
	Hui nationality	118	7.32
	Other nationality	109	6.76
Age		32.78 ± 14.03	
Work year		10.13 ± 7.27	
Educational level	College and lower	835	51.77
	Undergraduate	760	47.12
	Master and above	18	1.11
Occupational department	Internal medicine department	853	52.88
	Surgery department	608	37.69
	Emergency medicine department	76	4.71
	Intensive care unit department	76	4.71
Professional title	Primary title	564	34.97
	Intermediate title	586	36.33
	Senior title	463	28.7

### Descriptive analysis of psychological violence, resilience, and empathy

Table 2 presented the descriptive statistics results of psychological violence, empathy, and resilience. A total of 534 nurses experienced psychological violences with mean score of ≥ 1 on WPVBI scale, which yielded a positive rate of 33.1% among nurses (as showed in Table 2).

**Table 2:** The score of psychological violence, resilience, and empathy

Scale	Dimension	M ± SD
WPVBI scale	Total score	28.72 ± 28.3
	Isolation from work	11.32 ± 9.89
	Attacks on professional status	8.89 ± 9.25
	Attacks on personality	4.45 ± 6.31
	Direct negative behavior	4.06 ± 5.24
CD-RISC scale	Total score	67.06 ± 18.35
	Tenacity	34.48 ± 10.26
	Strength	22.12 ± 6.05
	Optimism	10.46 ± 3.08
JSE-HP scale	Total score	25.35 ± 17.52
	Perspective-taking	13.58 ± 9.11
	Compassionate care	3.94 ± 6.69
	Standing in patient’s shoes	7.84 ± 3.22

### Correlation analysis of psychological violence, resilience, and empathy

Table 3 presented the Pearson analysis results of psychological violence, empathy, and resilience. Pearson analysis reported a negative correlation between psychological violences and empathy (*r*=-0.724, *P* < 0.01) as well as a negative correlation between psychological violences and resilience (*r*=-0.681, *P* < 0.01) (as showed in Table 3).

**Table 3:** The correlation analysis of psychological violence, empathy, and resilience

Variables	1	2	3
1. Psychological violence	1		
2. Resilience	-0.681***	1	
3. Empathy	-0.724***	0.626***	1

*P < 0.05; **P < 0.01; ***P < 0.001

### Mediation analysis of psychological violence, resilience, and empathy

Table 4 presented the mediation analysis results of resilience among the relationship between psychological violence and empathy (as showed in Table 4). Model 4 in SPSS macro program PROCESS was used to examine the mediation role of resilience between psychological violence and empathy, psychological violence was the independent variable (X), resilience was the mediation variable (M), and empathy was the dependent variables (Y). Mediation analysis found that psychological violence negatively predicted resilience, a=-0.37, SE = 0.01, *p* < 0.001; both psychological violence and resilience entered the regression equation, psychological violence negatively predicted empathy, c’= -0.36, SE = 0.01, *P* < 0.001, resilience positively predicted empathy, b = 0.30, SE = 0.03, *p* < 0.001.

Bootstrap method found that resilience partially mediated the negative relationship between psychological violence and empathy, ab=-0.11, Boot SE = 0.03, 95% confidence interval [0.23, 0.36].

**Fig. 1:**
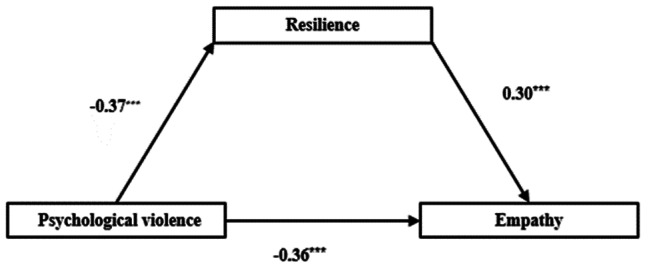
The mediation model of resilience among the relationship between psychological violence and empathy

The mediation effect accounted for ab/ (ab + c’) = 23.40% -0.37*0.30/ (-0.37*0.30+-0.36) = 23.4% of the total effect (as shown in Table 4; Fig. 1).

**Table 4:** The mediation model of resilience among the relationship between psychological violence and empathy

Variables	Resilience			Empathy		
	β	SE	t	β	SE	t
Psychological violence	-0.37	0.01	-37.32^***^	-0.36	0.01	-24.48^***^
Resilience				0.30	0.03	10.95^***^
*R* ^*2*^	0.46			0.56		
*F*	1393.07^***^			1010.89^***^		

## Discussion

Workplace psychological violence represents a pervasive public health problem with worldwide concern among healthcare workers, nurses represent a profession which faces the higher risk of exposure to workplace psychological violences among healthcare workers [2]. The present cross-sectional study recruited a convenience sample of 1613 clinical registered nurses to investigate the status of psychological violence, empathy, and resilience among nurses, explore the relationships of psychological violence, empathy, and resilience among nurses, and test the mediation effect of resilience among the relationship between psychological violence and empathy.

This survey recruited a convenience sample of 1613 clinical registered nurses with diversity in demographic characteristics in terms of ethnicity, age, work years, educational level, occupational department, and professional title. Thus, this convenience sample of 1613 clinical registered nurses at the provincial level constituted a representative snapshot of nurses across the national level. The present survey recruited a female dominant sample, which aligned with the patriarchal system in Chinese culture shaped nursing as a female career and providing care as a female job. The National Health Commission in China supported nurse as a female dominant profession since male constituted only 3% of Chinese registered nurses at the end of the year 2021 [35].

This study found that about 33.1% of clinical registered nurses experienced workplace psychological violences in the past year, which was consistent with previous studies. A cross-sectional study of 1,761 nurses from 9 public tertiary hospitals in 4 provinces reported a prevalence of 59.64% for workplace psychological violences among Chinese nurses [36]. A cross-sectional study examined the prevalence of workplace psychological violences among emergency nurses in China, which recruited a convenience sample of 243 emergency nurses from 10 tertiary hospitals in Beijing, found that 63.3% of emergency nurses experienced psychological violences [3]. A cross-sectional study in southwest China investigated the prevalence of workplace psychological violences against healthcare professionals in a multiethnic area, reported a 5.5% prevalence of physical violences and a 43.7% prevalence of psychological violences among 2036 healthcare professionals in the previous 12 months [37]. The prevalence of workplace psychological violences against nurses in this study fall within the range of prevalence in previous studies which investigated the prevalence of workplace psychological violences against nurses in various clinical departments across diverse geographical regions under the context of Chinese healthcare systems [3, 36, 37]. Previous investigation of workplace psychological violences against nurses in China mainly conducted at provincial levels, which failed to present an accurate picture of workplace psychological violence incidence against nurses in China at national level [38–40]. The prevalence of workplace psychological violences against nurses varied across different studies due to diverse perceptions of psychological violence definition and various measures of psychological violence scales among different studies [8]. The high prevalence of workplace psychological violences urgent the prevention and management of workplace psychological violences at hospital and individual levels. The Chinese State Council promulgated the Regulations on Prevention and Treatment of Medical Disputes in 2018 strengthened the administration of workplace violence and the prevention of workplace violence in policies and regulations.

Pearson analysis reported an inverse correlation between workplace psychological violences and empathy, which implicated that exposure to workplace psychological violences negatively predicted empathy. The findings from this study coincided with findings from previous studies which reported a negative correlation between workplace psychological violences and empathy. Empathy refers to a cognitive attribute which allows nurses to insight, perceive, and experience the concerns, emotions, and perspectives of patients [41, 42]. Exposure to workplace psychological violences not only threatened the health and safety of nurses, but also caused psychological distress and emotional frustration for nurses. Nurses who exposure to workplace psychological violences reduced passions to work and decreased concentrations on care, thus impaired empathy ability and hindered work performance. Lu et al. (2022) preformed a cross-sectional survey to explore the relationship between workplace psychological violences and empathic competence in nurse, the investigation recruited a total of 1954 Chinese nurses from 4 tertiary hospitals in Shandong province, linear regression found that psychological violences was negatively correlated with empathy ability in nurses [43]. Zhang et al. (2017) performed a cross-sectional survey of 3835 Chinese nurses from 28 hospitals, found that nurses with lower level of empathy exhibited higher likelihood of workplace violence [44]. The present study in conjunction with previous studies demonstrated that the experience of workplace psychological violences impaired the empathy of victimized nurses, which emphasized the development of prevention programs and intervention strategies to manage the destructive effects of workplace psychological violences on nurses. 

Pearson analysis reported an inverse correlation between workplace psychological violences and resilience, which implicated that exposure to workplace psychological violences negatively predicted resilience. The present study aligned with previous studies which reported a negative correlation between workplace psychological violences and resilience. Nurses experienced workplace psychological violences not only negatively tolerated adverse consequences but also actively mobilized personality resources and psychological capitals to resist psychological violences. Resilience represents a personality resource and psychological capital which enables nurses to copy with pressure events and adapt to stressful contexts in positive manners [45, 46]. Nurses equipped with resilience adopted positive reactions to violences and taken optimistic view toward violences, thus alleviated psychological distress and emotional stress after exposure to psychological violences. Fan et al. (2022) conducted an across-sectional study which involved 349 nurses to explore the relationship between workplace violence and resilience, correlation coefficient analysis found that workplace violences was negatively associated with resilience [21]. Itzhaki et al. (2015) conducted an across-sectional study which recruited 118 mental health nurses to explore the relationship between workplace violences and resilience, Pearson correlation coefficients found that verbal violences and physical violences were negatively associated with resilience [47]. The findings of this study in concert with findings from previous studies demonstrated that the presence of resilience alleviated the impact of psychological violences among these nurses, which provided theoretical basis for prevention and intervention formulation to resist the destructive effects of psychological violences on nurses. 

Mediation analysis supported the mediation effect of resilience among the negative relationship between workplace psychological violences and empathy. Resilience represents one of the most important protective factors which equips nurses with psychological resources and personality capacities to copy with and adapt to psychological violences [48]. Resilience enables nurses to mobilize resources to address workplace adversity and adopt strategies to minimize psychological distress after exposure to psychological violences [49]. Previous studies in line with the present study supported a mediation role of resilience on the relationship between workplace violences and nurse outcomes [21, 50]. The protective factor model of resilience stated that resilience could reduce the cumulative effects of risk factors on negative outcomes, which further supported that resilience as a protective factor could mediated the negative effects of workplace psychological violences on empathy [51].

### Limitation

The self-reported measure of this survey could cause a risk of recall/report bias due to inaccurate responses. Further studies should combine multiple sources of information or/and multiple methods of data collection to overcome the subjective bias from self-reports. The cross-sectional design of this study could preclude a causal inference of these variables. Further studies with experimental and longitudinal designs should be performed to explore more complex interactions among these variables.

## Conclusion

Workplace psychological violence refers to any act of psychological terrors which ranged from verbal abuses to verbal tortures directed toward individual at work or on duty. Workplace psychological violence constitutes both a serious occupational hazard for healthcare workers and a major global concern for healthcare systems. Nurses are the most vulnerable occupational group for workplace psychological violences in healthcare systems, who are subject to psychological violences from colleagues, leaders, and patients in workplaces [52, 53]. Nurses who exposure to workplace psychological violences experience a series of adverse consequences which includes decreased organization commitment and profession satisfaction as well as increased emotional exhaustion and psychological distress. The prevalence and seriousness of workplace psychological violences among nurses urgent the characterization of negative impacts and protective factors across diverse healthcare systems and cultural contexts. Therefore, the present cross-sectional study recruited a convenience sample of 1613 clinical registered nurses to investigate the status of psychological violences, empathy, and resilience among nurses, and explore the relationships of psychological violences, empathy, and resilience among nurses. The present study supported an inverse relationship between psychological violences and empathy among Chinese nurses where resilience mediated the negative impacts of psychological violences on empathy. Nurses who exposure to workplace psychological violences decreased their empathic ability where resilience acted as a protective factor to buffer the negative impacts of psychological violences on empathy.

## Relevance for clinical practice

Workplace psychological violence constitutes a topic of public health issue and academic research attention, which impairs the personal lives and professional works of nurses as well as increases job dissatisfaction and turnover intention of nurses. Nurses who exposure to workplace psychological violences decreased their empathic ability where resilience acted as a protective factor to buffer the negative impacts of psychological violences on empathy. The present study highlighted the seriousness of workplace psychological violences against nurses and emphasized the importance of resilience in nurses, thus provided theoretical basis for health policies and clinical interventions where equip nurses with resilience to copy with and recover from workplace psychological violences.

## Data Availability

The data that support the findings of this study are available from the corresponding author Dr. Xiaoli Liao upon reasonable request.
